# Ethnicity and pathways to care during first episode psychosis: the role of cultural illness attributions

**DOI:** 10.1186/s12888-015-0665-9

**Published:** 2015-11-16

**Authors:** Swaran P. Singh, Luke Brown, Catherine Winsper, Ruchika Gajwani, Zoebia Islam, Rubina Jasani, Helen Parsons, Fatemeh Rabbie-Khan, Max Birchwood

**Affiliations:** Mental Health and Well Being, Warwick Medical School, University of Warwick, Coventry, CV4 7AL UK; LOROS, Hospice Care for Leicester, Leicestershire and Rutland, Leicester, UK; De Montfort University, Leicester, UK; Institute of Health and Wellbeing, University of Glasgow, Glasgow, UK; HCRI, University of Manchester, Manchester, UK; Faculty of Health, Education & Life Sciences, Birmingham City University, Birmingham, UK; Division of Health Sciences, Warwick Medical School, University of Warwick, Coventry, UK

**Keywords:** Ethnicity, Illness attributions, Compulsory detention, Early intervention, First episode psychosis

## Abstract

**Background:**

Studies demonstrate ethnic variations in pathways to care during first episode psychosis (FEP). There are no extant studies, however, that have statistically examined the influence of culturally mediated illness attributions on these variations.

**Methods:**

We conducted an observational study of 123 (45 White; 35 Black; 43 Asian) patients recruited over a two-year period from an Early Intervention Service (EIS) in Birmingham, UK. Sociodemographic factors (age; sex; education; country of birth; religious practice; marital status; living alone), duration of untreated psychosis (DUP), service contacts (general practitioner; emergency services; faith-based; compulsory detention; criminal justice) and illness attributions (“individual;” “natural;” “social;” “supernatural;” “no attribution”) were assessed.

**Results:**

Ethnic groups did not differ in DUP (*p =* 0.86). Asian patients were more likely to report supernatural illness attributions in comparison to White (Odds Ratio: 4.02; 95 % Confidence Intervals: 1.52, 10.62) and Black (OR: 3.48; 95 % CI: 1.25, 9.67) patients. In logistic regressions controlling for confounders and illness attributions, Black (OR: 14.00; 95 % CI: 1.30, 151.11) and Asian (OR: 13.29; 95 % CI: 1.26, 140.47) patients were more likely to consult faith-based institutions than White patients. Black patients were more likely to be compulsorily detained than White patients (OR: 4.56; 95 % CI: 1.40, 14.85).

**Conclusion:**

Illness attributions and sociodemographic confounders do not fully explain the ethnic tendency to seek out faith-based institutions. While Asian and Black patients are more likely to seek help from faith-based organisations, this does not appear to lead to a delay in contact with mental health services.

## Background

The early detection and management of psychosis through specialist Early Intervention Services (EIS) is one of the most important service developments in mental health care in the past two decades [1]. A clear understanding of the pathways to care during first episode psychosis (FEP) is especially important as initial experiences and interactions may have lasting impact on subsequent help-seeking, service engagement and adherence to treatment. Pathways to care during FEP can be influenced by social, cultural, and health service factors [2]. Ethnicity, in particular, may influence care pathways by impacting on explanatory models of illness, social connections, and help seeking behaviour [3]. In a recent meta-analysis of ethnic variations in pathways to care during FEP, Anderson, Flora [3] found that Black (but not Asian) patients were significantly less likely to have general practitioner (GP) involvement, and significantly more likely to experience police contact, in comparison to White patients. The determinants of these, and other, ethnic variations in pathways to care during FEP remain largely untested [3, 4].

Studies examining ethnic variations in illness attributions and subsequent help seeking in early psychosis are sparse, and to the best of our knowledge there are no studies with FEP populations in the UK. Though not specific to psychosis, Hatfield, Mohamad [5] found that Asian mental health service users and members of the Asian population in the UK reported prominent religious attributions for the causes and treatment of mental illness. In a quantitative study with chronic schizophrenia patients, McCabe and Priebe [6] found that White patients were significantly more likely to attribute their symptoms to biological causes than non-white (i.e., Bangladeshis, African-Caribbeans and West Africans) patients, who were significantly more likely to cite supernatural causes. Of note, a biological model of illness was significantly associated with enhanced treatment satisfaction and therapeutic relationships [6]. As this study did not adjust for relevant confounders (e.g., religion; education; country of birth), it is difficult to draw conclusions regarding the independent effects of ethnicity and culturally mediated illness models on service experience.

Small qualitative studies from outside the UK have suggested that supernatural explanatory models of illness may lead to help-seeking from traditional faith healers rather than mental health services [7–9]. The relevance of these studies to the UK, however, is unclear as associations are likely confounded by cultural context and the availability of mental health services in different countries. The extent to which cultural illness attributions may lead to faith-based help seeking in the UK is an important question as it has been suggested that faith-based help seeking may lead to longer duration of untreated psychosis [10], which may subsequently worsen prognosis and relapse rates [11].

The main aim of the current study was to examine whether culturally-mediated illness attributions (e.g., individual, supernatural) and related confounders (e.g., religious practice) influence ethnic variations in pathways to care (e.g., compulsory detention, general practitioner, faith-based organisations) during FEP. Specifically, we examined the following questions:

1. Do ethnic groups significantly differ in culturally mediated illness attributions (i.e., individual, natural, social, supernatural, and no attribution) during FEP?
2. Do ethnic groups significantly differ in their pathways to care (i.e., DUP, compulsory detention, criminal justice contact, emergency services, general practitioner, faith-based organisations) during FEP?
3. Are ethnic pathways to care during FEP influenced by culturally mediated illness attributions and other relevant sociodemographic (i.e., gender, religious practice, marital status, living alone, country of birth, education) and clinical (duration of untreated psychosis) confounders?

## Methods

### Participants

The study was part of the NIHR funded ENRICH Programme Grant which aimed to explore ethnic differences in access to mental health care in FEP [12]. Methodological details including the base population, case ascertainment, recruitment and assessment have been reported in detail elsewhere [12]. Briefly, patients were recruited over a two-year period (2008–2010) from the Early Intervention Service (EIS) of the Birmingham and Solihull Mental Health Foundation Trust (BSMHFT). BSMHFT is one of the largest mental health Trusts in the UK and provides comprehensive mental health care to a population in excess of 1 million. All participants were recruited from the Birmingham EIS; the Solihull EIS service was not established at study commencement. Each eligible participant’s Community Psychiatric Nurse (CPN) was approached to determine whether the patient was well enough to take part in the study. If the CPN felt that the patient was suitable, they gave them an information sheet and consent form. If the patient agreed to meet the research team, a researcher contacted the patient to explain the study and answer any questions. Of the consecutive cases who were approached (*n* = 499), 132 (45 White, 35 Black, 43 Asian, and 9 “other;” age range: 14 to 37 years) took part in the study. Written consent was obtained from adult patients, and parental written consent was obtained for patients who were classed as minors. The most common reasons for non-participation were: the patient was not interested in the research; the patient was not able to give informed consent; the patient was not engaging clinically with services. A demographic comparison of patients entering the EIS across the two year recruitment period did not indicate any notable variations in ethnic distribution, age or gender between those who did and did not consent [12]. Sociodemographic and clinical details were collected and patients were interviewed about illness attributions and service encounters occurring during the prodromal and psychotic phase of their illness. The study received full ethical approval from Warwickshire Research Ethics Committee (WREC). Once ethical approval was granted, the study was reviewed and approved prior to commencement by BSMHFT Research and Innovation Department. In the current study we focus on patient-reported illness attributions and service encounters occurring during the psychotic phase of the illness.

### Instruments

#### Ethnicity

Ethnicity was assessed in two ways. First, participants were asked to describe their ethnicity in their own words. Second, a list of census categories was presented to participants, who were asked to select the category that best represented their ethnic group. Responses were consistent across methods. We recoded ethnicity according to the following four categories: Asian (i.e., Bangladeshi, Indian, Pakistani); Black (African, Caribbean); White (British, Irish) and “other” (other, mixed other, mixed White and Black Caribbean) [13]. These higher order categories were constructed to afford statistical power for analysis. We did not include the “other” patient group in the analyses due to the limited number of cases (*n* = 9).

#### Service encounters interview

Service encounters during the psychotic phase of the illness were assessed with an amended version of the *Encounter form* by Gater, Sousa [14] to increase relevance to the current study. Main amendments included the incorporation of additional services (e.g., early intervention services and Child and Adolescent Mental Health Services) and questions pertaining to the phase of illness (i.e., prodromal versus psychotic). All medical notes and correspondence were collated into a timeline detailing each patient’s journey to psychiatric care. This information was presented to the patient and carer-informant for confirmation, and so that they could describe any other help-seeking avenues (e.g., faith based encounters) they may have experienced. Encounter types comprised the following: general practitioner (GP)/accident and emergency (A & E), mental health service, welfare service, criminal justice contact, compulsory detention, and faith-based organisations (e.g., spiritual leader from a local mosque). Each type of service encounter was categorised in two ways for the analysis: 1) as a percentage of the total number of service encounters during the psychotic phase (e.g., 4 faith based contacts out of 10 total contacts = 40 %); 2) as a dichotomous variable representing at least one encounter during the psychotic phase for each service type (e.g., 0 = no faith based contact; 1 = at least one faith based contact).

#### Illness attribution scale

*The Emerging Psychosis Attribution Schedule* (EPAS) is a semi-structured interview used to assess patient and carer attributions of symptoms in the emerging phase of a psychotic episode. It is based on the Short Explanatory Model Interview (SEMI) [15] with amendments to reduce medical terminology and incorporate assessment of symptom attribution over time. Responses were categorised into four main domains [16]: *individual* (e.g., psychological or physiological causes); *natural* (e.g., as a result of germs, toxins or a reaction to accidents/drugs, etc.); *social* (e.g., social experiences and adverse events); and *supernatural* (e.g., spiritual possession or supernatural punishment). Following a pilot, a fifth domain was added representing *no attribution* for psychotic symptoms (i.e., the patient was unaware of disorder or gave no attribution of causation). The inter-rater reliability of the EPAS was assessed with 15 randomly selected transcripts of the symptom attribution interviews. Inter-rater agreement was good between researchers, achieving a kappa coefficient of 0.76 across all elicited attributions. Each attribution domain (i.e., individual, supernatural, social, natural, no attribution) was categorised in two ways for analysis: 1) as a percentage of total number of illness attributions for each participant (as described for service encounters above); 2) as a dichotomous variable indicating the presence of at least one relevant attribution (e.g., 0 = no supernatural attribution; 1 = at least one supernatural attribution).

#### Duration of untreated psychosis (DUP)

The Nottingham Onset Schedule (NOS) was used to establish the timing of onset of the psychotic illness. The NOS is a short, guided interview and rating scale that records the details of the components of onset of a psychotic illness. The NOS has high test-retest and inter-rater reliability [17] and is a standard measure for DUP in several early intervention services [18]. The NOS defines onset as comprising of three illness phases: a) a prodrome phase (i.e., between onset of prodrome and definite diagnosis); b) duration of untreated psychosis (DUP) (i.e., between definite diagnosis and treatment compliance); and c) duration of untreated illness (DUI) (i.e., between onset of prodrome and treatment compliance). For the current study, we were interested in DUP. In line with previous research [19], we categorised DUP as short (≤6 months) and long (>6 months).

#### Sociodemographic confounders

##### Living status

Participants were asked if they lived: “alone,” “with parents/guardians,” “with a partner,” “alone with children,” or “other.” Responses were recoded dichotomously as: 0 = lives with others; 1 = lives alone.

##### Marital status

Participants were asked what their current marital status was, which was coded dichotomously as: 0 = married/cohabiting; 1 = single.

##### Country of birth

Participants reported their birth place (i.e., Africa, Caribbean, South Asian, UK, Other), which was recoded as: 0 = UK; 1 = outside of the UK.

##### Education status

Education status was classified as: 0 = to school level; 1 = beyond school level (i.e., higher education).

##### Religion

Participants were asked to describe their faith (i.e., Christian, Sikh, Catholic, Muslim, Hindu, Atheism, Agnostic, Spiritual, Other and None) and whether they practiced this religion. We coded participant responses into: 0 = does not practice religion; 1 = practices religion.

### Statistical analysis

We conducted the analysis in three stages using *SPSS version 22* [20]. First, we used *x*^*2*^ tests to compare ethnic groups on sociodemographic (e.g., education, living status) and clinical (e.g., DUP, diagnosis) factors (reported in Table 1). Second, we tested unadjusted associations between ethnic groups and culturally mediated illness attributions (reported in Table 2) and service encounters (reported in Table 3) with ANOVAs (for approximately normally distributed continuous outcomes), Kruskal-Wallis rank sum tests (for non-normally distributed continuous outcomes) and logistic regressions (for dichotomous outcomes). Third, we utilised multiple logistic regression analysis (reported in Table 4) to test adjusted associations between ethnicity and service encounters (dichotomously coded). In Model A we controlled for sociodemographic and clinical confounders (i.e., age, gender, country of birth, DUP, religious practice, education, marital and living status) and in Model B we additionally controlled for culturally mediated illness attributions (i.e., individual, natural, social, supernatural and no attribution).

**Table 1:** A sociodemographic and clinical comparison of White, Black and Asian FEP patients

Sociodemographic factor	Total	White	Black	Asian	*P* value
	*n* = 123	*n* = 45	*n* = 35	*n* = 43	
*Gender*					
Female	32 (26 %)	9 (20 %)	11 (31.4 %)	12 (27.9 %)	0.48
Male	91 (74 %)	36 (80 %)	24 (68.6 %)	31 (72.1 %)	
*Age (Mean, SD)*	23.22 (5.07)	23.13 (4.63)	22.71 (4.69)	23.72 (5.83)	0.68^a^
*Education*					
School	64 (52.0 %)	22 (48.9 %)	20 (57.1 %)	22 (51.2 %)	0.757
Higher	59 (48.0 %)	23 (51.1 %)	15 (42.9 %)	21 (48.8 %)	
*Religious affiliation*					
Christianity	44 (35.8 %)	15 (33.3 %)	29 (82.9 %)	0 (0 %)	
Other	4 (3.3 %)	0 (0 %)	1 (2.9 %)	3 (7 %)	**<0.001**
Islam	38 (30.9 %)	1 (2.2 %)	1 (2.9 %)	36 (83.7 %)	
None	37 (30.1 %)	29 (64.4 %)	4 (11.4 %)	4 (9.3 %)	
*Religious practice*					
No	65(52.8 %)	38 (84.4 %)	16 (45.7 %)	11 (25.6 %)	**<0.001**
Yes	58(47.2 %)	7 (15.6 %)	19 (54.3 %)	32 (74.4 %)	
*Birth place*					
UK	100 (81.3 %)	44 (97.8 %)	23 (65.7 %)	33 (76.7 %)	**<0.01**
Non UK	23 (18.7 %)	1 (2.2 %)	12 (34.3 %)	10 (23.3 %)	
*Migrant generation*					
1^st^ Generation	27 (22 %)	1 (2.2 %)	13 (37.1 %)	13 (30.2 %)	
2^nd^ Generation	32 (26 %)	0 (0 %)	8 (22.9 %)	24 (55.8 %)	**<0.001**
3^rd^ Generation	21 (17.1 %)	1 (2.2 %)	14 (40 %)	6 (14 %)	
Not applicable	43 (35 %)	43 (95.6 %)	0 (0 %)	0 (0 %)	
*Marital status*					
Married/cohabiting	13 (10.6 %)	3 (6.7 %)	1 (2.9 %)	9 (20.9 %)	**0.02**
Single	110 (89.4 %)	42 (93.3 %)	34 (97.1 %)	34 (79.1 %)	
*Living status*					
Alone	29 (23.6 %)	8 (17.8 %)	17 (48.6 %)	4 (9.3 %)	**<0.001**
With others	94 (76.4 %)	37 (82.2 %)	18 (51.4 %)	39 (90.7 %)	
*DUP*					
≤ 6 months	48 (39.4 %)	19 (42.2 %)	13 (37.1 %)	16 (37.2 %)	0.859
> 6 months	75 (61.0 %)	26 (57.8 %)	22 (62.9 %)	27 (62.8 %)	
*Diagnosis*					
Depressive psychosis	30 (25.6 %)	14 (36.2 %)	7 (20.6 %)	9 (22.5 %)	
Broad schizophrenia	84 (71.8 %)	27 (62.8 %)	27 (79.4 %)	30 (75 %)	0.446
Manic psychosis	3 (2.6 %)	2 (4.7 %)	0 (0 %)	1 (2.5 %)	

Results based on chi square analysis; *DUP* Duration of Untreated Psychosis; ^a^Results based on ANOVA as continuous outcome

Bold typeface indicates significant group difference

**Table 2:** Ethnic variations in proportional and dichotomous illness attributions during FEP

Illness attribution	White	Black	Asian	*P* value	Odds ratio (95 % CI)^c^	
Individual	14.07%^a^	10.71 %	13.57 %	0.572^b^	White vs Asian	0.94 (0,35, 2.50)
					White vs Black	0.64 (0.21, 1.94)
					Black vs Asian	1.47 (0.47, 4.53)
Natural	4. 89 %	8.10	4.26 %	0.443^b^	White vs Asian	1.05 (0.20, 5.51)
					White vs Black	2.33 (0.52, 10.52)
					Black vs Asian	0.45 (0.10, 2.03)
Social	6.48 %	5.95 %	2.33 %	0.373^b^	White vs Asian	0.32 (0.06, 1.67)
					White vs Black	0.84 (0.22, 3.24)
					Black vs Asian	0.38 (0.07, 2.20)
Supernatural	7.0 %	11.9 %	34.11 %	**0.001** ^b^	White vs Asian	**4.02 (1.52, 10.62)**
					White vs Black	1.16 (0.38, 3.57)
					Black vs Asian	**3.48 (1.25, 9.67)**
No attribution	54.22 %	54.76 %	29.46 %	**0.009** ^b^	White vs Asian	**0.33 (0.14, 0.78)**
					White vs Black	0.99 (0.38, 2.56)
					Black vs Asian	**0.33 (0.13, 0.84)**

*CI* Confidence Interval; ^a^Mean group percentage of total attributions; ^b^Based on the Kruskall-Wallis test for non-normally distributed data; ^c^Based on logistic regression analysis with at least 1 attribution as the outcome, Bold typeface indicates significant Odds Ratio

**Table 3:** Ethnic variations in proportional and dichotomous service encounters during FEP

Service encounters	Total	White	Black	Asian	*P* value	Odds ratio (95 % CI)^c^	
Mental health services	40.73 %	40.90 %	41.95 %	39.56 %	0.831^a^	N/A	N/A
Emergency services/general	19.99 %	22.61 %	19.17 %	17.93 %	0.465^b^	White vs. Asian	0.38 (0.14, 1.00)^**d**^
Practitioner						White vs. Black	1.56 (0.64, 3.81)
						Asian vs. Black	**4.13 (1.50, 11.40)**
Welfare services	3.64 %	3.16 %	5.38 %	2.74 %	0.257^b^	White vs. Asian	0.71 (0.21, 2.45)
						White vs. Black	1.88 (0.62, 5.68)
						Asian vs. Black	2.63 (0.79, 8.75)
Faith based services	3.51 %	0 %	3.27 %	7.39 %	**<0.001** ^**b**^	White vs. Asian	**34.83 (4.39, 279.46)** ^**e**^
						White vs. Black	**13.04 (1.54, 110.07)**
						Asian vs. Black	0.37 (0.14, 1.01)
Criminal justice	6.34 %	4.46 %	8.36 %	6.66 %	0.121^b^	White vs. Asian	1.49 (0.59, 3.79)
						White vs. Black	**2.60 (1.01, 6.74)**
						Asian vs. Black	1.74 (0.69, 4.38)
Compulsory detention	N/A	N/A	N/A	N/A	N/A	White vs. Asian	1.52 (0.58, 3.95)
						White vs. Black	**4.67 (1.77, 12.32)**
						Asian vs. Black	**3.08 (1.21, 7.83)**

Columns 2 to 5 represent the proportion of total FEP encounters for each ethnicity (columns do not equal 100 % as not all encounters [e.g., EI] are reported here); Column 8 represents at least one service encounter for each type of service; *CI* Confidence Interval, *N/A* data not available/applicable; ^a^Based on ANOVA test; ^b^Based on Kruskall-Wallis test; ^c^Logistic regression with at least one encounter as the outcome; ^d^At least one emergency service encounter (excluding GP contact); ^e^A value of 1 was added to each cell to facilitate logistic regression as there were no contacts for White patients

Bold typeface indicates significant Odds ratio

**Table 4:** Ethnic variations in service encounters during FEP adjusting for confounders and illness attributions

Ethnicity	Compulsory detention		Criminal justice contact		Faith based ^a^		Emergency services		General practitioner	
	Odds ratio	*p*	Odds ratio	*p*	Odds ratio	*p*	Odds ratio	*p*	Odds ratio	*p*
W vs B	**4.46 (1.40, 14.18)** ^**b**^	**0.011**	2.87 (0.89, 9.32)^b^	0.079	**18.80 (1.93, 183.28)** ^**b**^	**0.012**	1.64 (0.55, 4.88)^b^	0.375	0.64 (0.20, 2.04)	0.453
	**4.56 (1.40, 14.85)** ^**c**^	**0.012**	3.06 (0.90, 10.42)^c^	0.074	**14.00 (1.30, 151.11)** ^**c**^	**0.030**	1.64 (0.52, 5.20)^c^	0.401	0.57 (0.17, 1.86)	0.351
W vs A	2.22 (0.70, 7.09)^b^	0.177	2.18 (0.67, 7.03)^b^	0.194	**30.41 (3.41, 270.98)** ^**b**^	**0.002**	0.31 (0.10, 1.02)^b^	0.054	0.70 (0.23, 2.15)	0.531
	2.79 (0.80, 9.73)^c^	0.107	3.21 (0.88, 11.67)^c^	0.076	**13.29 (1.26, 140.47)** ^**c**^	**0.031**	0.37 (0.10, 1.38)^c^	0.138	0.67 (0.20, 2.27)	0.518
A vs B	2.01 (0.69, 5.81)^b^	0.200^a^	1.32 (0.43, 4.02)^b^	0.625	0.62 (0.19, 1.97)^b^	0.416	**5.25 (1.61, 17.15)** ^**b**^	**0.006**	0.92 (0.30, 2.80)	0.885
	1.63 (0.53, 5.04)^c^	0.393	0.95 (0.29, 3.16)^c^	0.936	1.05 (0.26, 4.30)^c^	0.942	**4.41 (1.25, 15.62)** ^**c**^	**0.021**	0.85 (0.26, 2.83)	0.793

*W* White, *B* Black, *A* Asian; ^a^A value of 1 was added to each cell to facilitate logistic regression as there were no contacts for White patients ^b^Adjusted for age, sex, DUP; marital status, UK birth, living alone, education status, religious practice; ^c^Further adjusted for previously listed confounders and culturally mediated illness attributions, Bold typeface indicates significant Odds Ratios

## Results

### Sociodemographic and clinical characteristics according to ethnic group

See Table 1 for a statistical comparison of sociodemographic and clinical characteristics between ethnic groups. Ethnic groups significantly differed in religious affiliation and practice (Asian patients, predominantly of Islamic faith, were most likely to practice religion), birth place (more White patients were born in the UK), marital status (Asian patients were more likely to be married than Black or White patients), and living status (Black patients were more likely to live alone). Ethnic groups did not significantly differ in DUP length (*p* = 0.86).

### Ethnic variations in illness attributions

There were some differences in culturally mediated illness attributions between ethnic groups (see Table 2). Asian patients were significantly more likely to report at least one supernatural attribution (*p* = 0.001), and significantly less likely to report no attribution (*p* = 0.009) for their symptoms in comparison to White or Black patients. Proportionally, supernatural illness attributions were the most common attribution in Asian patients, while no attribution for symptoms (i.e., the patient was unaware of disorder or gave no attribution of causation) was the most common attribution in Black and White patients.

### Illness attributions as predictors of service encounters

Supernatural illness attributions significantly predicted faith based encounters: OR = 2.54; 95 % CI: 1.07, 5.99 (*p* = 0.034), while natural illness attributions significantly predicted accident and emergency service encounters: OR = 3.82; 95 % CI: 1.06, 13.86 (*p* = 0.041).

### Ethnic variations in service encounters

Asian patients utilised a significantly (*p* <0.001) higher proportion of faith-based support than White or Black patients. Asian (*p* = 0.001) and Black (*p* = 0.018) patients were significantly more likely to have at least one faith-based contact in comparison to White patients. Asian patients were significantly less likely to have at least one emergency service contact. Black patients were significantly more likely to be compulsorily detained and experience criminal justice contact (at least once) in comparison to White (*p* = 0.002) and Asian (*p* = 0.018), and White (*p* = 0.049), patients respectively (see Table 3). As a proportion of total FEP encounters, mental health service encounters were most common for all ethnicities.

Following control for confounding factors, differences in compulsory detention between Black and White patients remained robust (and little attenuated). Asian patients remained less likely to have contact with emergency services than Black patients. Asian (*p* = 0.031) and Black (*p* = 0.030) patients remained significantly more likely to consult faith-based healers; though associations (for Asian patients) were considerably attenuated following adjustment for culturally mediated illness attributions (see Table 4).

## Discussion

Our study is unique in examining ethnic variations in the care pathways of FEP patients and how they are influenced by culturally mediated illness attributions and other associated confounders. In relation to our research questions, we have three main findings. First, Asian patients were significantly more likely to attribute their psychotic symptoms to supernatural causes in comparison to White and Black patients, while Black and Asian patients reported a significantly higher proportion of faith-based encounters in the help-seeking pathway in comparison to White patients. Second, Black patients were significantly more likely to be compulsorily detained than White patients. Third, ethnic differences in pathways to care during FEP were largely unaltered (i.e., remained significant) by culturally mediated illness attributions and associated confounders, and there were no ethnic variations in DUP or proportion of mental health service encounters. We will discuss these findings in turn.

The finding that Asian patients were significantly more likely to attribute their psychotic symptoms to supernatural causes is largely consistent with previous studies in the UK [5, 6] and extends this work by demonstrating that Asian patients in particular are more likely to attribute psychotic symptoms to supernatural causes during FEP. Similar to findings from studies outside of the UK [7–9], we found that supernatural illness attributions significantly predicted faith-based contact, and that incorporating these attributions into the final model considerably attenuated the association between Asian ethnicity and faith-based encounters (though this association remained significant). Conversely, associations between Black ethnicity and faith-based encounters were not substantially attenuated by supernatural attributions and confounders. These findings indicate that the influence of supernatural illness attributions and associated confounders may differ across different ethnicities. Other potential drivers of faith-based help-seeking may include family and community influences [21] and service level factors, such as a lack of spiritual and cultural awareness training in EIS clinicians [21]. In a sub-set of the current sample, Asian patients reported receiving solace and benefit from faith-based organisations [21]. Nevertheless, spiritual care representatives had little contact with mental health services, which may be partly attributable to reluctance on the part of service users to disclose faith-based beliefs and practices to EIS staff [21].

Consistent with a previous study of four early intervention services across the UK [22], we found that compulsory detention was significantly more likely in Black than White or Asian patients. This increased risk was not explicable by illness attributions, or relevant confounders including: DUP, living alone, marital status, education, or country of birth. Mann, Fisher [22] concluded that EIS input in its current form has had little impact on reducing detention rates in Black minority groups. Indeed, our findings would appear to support this contention. As adverse contacts have been consistently linked to poorer outcomes in psychosis [23], this area merits further research attention [22].

Similar to a number of previous studies [24–26], we found that DUP did not significantly differ as a function of ethnicity. Other studies, however, have indicated ethnic variations in DUP, with some supporting longer DUP in White patients [10, 27]. Thus, it appears that the association between treatment delay and ethnicity is complex and may be indirect (i.e., mediated by other factors such as criminal justice service contact [27]). Of note, Bhui, Ullrich [10] reported longer DUP in FEP patients who had utilised faith-based support; however, findings were non-significant and only very few patients in the study had utilised faith-based support. Our study does not support that faith-based service utilisation is associated with longer DUP, but rather that Black and Asian patients are likely to use both faith-based and mental health services concurrently. This underscores the importance of increasing collaboration between EIS and faith-based organisations to ensure the delivery of holistic, person-centred care [21, 28].

Our study had some limitations. First, due to the in-depth nature of the assessments, our sample was relatively small, which will have reduced power potentially leading to type II errors. The small sample size precluded analysis of more finely grained ethnic (e.g., African versus Caribbean) and religious (e.g., Muslim versus Hindu) categories and did not allow for complex path models to explicitly examine the mediational role of illness attributions and confounders. Nevertheless, our study demonstrated subtle variations between Black and Asian care pathways on which to base future large scale studies. Second, our sample may have been subject to selection bias as recruitment rates were low (i.e., some service users were not well enough to give informed consent), which may have limited the generalisability of our findings. However, a demographic comparison of patients entering the EIS across the two-year recruitment period did not indicate any notable variations in ethnic distribution, age or gender between those who did and did not consent. Third, we developed the Emerging Psychosis Attribution Schedule (EPAS) in order to assess patient attributions of symptoms during the emerging phase of the psychotic illness. How closely each identified attribution domain, e.g., “individual” maps onto those designated in previous studies [6] remains unknown. The EPAS did, however, demonstrate good inter-rater agreement and took into account attributions during different stages of the disorder. Fourth, while we had details regarding migrant generation (i.e., 1^st^, 2^nd^ or 3^rd^ generation), we could not incorporate this variable into our main analysis as it was confounded by ethnicity (i.e., nearly all White patients were excluded by definition), and we did not have a sufficient sample size to stratify our analyses according to generational status. Future studies may consider the extent to which ethnic variations in illness attributions and care pathways differ as a function of migrant generation [29]. Finally, dates of illness phases were not exact as we relied on retrospective report of illness attributions and service encounters. Where possible, however, we corroborated patients’ accounts with medical notes and clinical correspondence.

## Conclusions

While Asian and Black patients are more likely to seek help from faith-based organisations, this does not appear to lead to a delay in contact with mental health services. Future studies may consider the moderating role of service level factors on ethnic variations in care pathways, and examine how services can develop innovative collaborative models to meet the individual needs of each patient [21].

## Abbreviations

ANOVA: Analysis of variance
FEP: First episode psychosis
DUP: Duration of untreated psychosis
EIS: Early intervention service
EPAS: Emerging psychosis attribution schedule
